# Obstetric Triage Scales; a Narrative Review 

**Published:** 2019-01-13

**Authors:** Farzaneh Rashidi Fakari, Masoumeh Simbar, Shahrzad Zadeh modares, Hamid Alavi Majd

**Affiliations:** 1 Students Research Committee, Department of Midwifery and Reproductive Health, School of Nursing and Midwifery, Shahid Beheshti University of Medical Sciences, Tehran, Iran. Email: Rashidiff@yahoo.com, orcid.org/0000-0001-7498-475X; 2 Midwifery and Reproductive Health Research Center, Shahid Beheshti University of Medical Sciences, Tehran, Iran. Email: msimbar@gmial.com , orcid.org/0000-0003-2843-3150; 3 Mahdiyeh Hospital Clinical Research Development Unit, Shahid Beheshti University of Medical Sciences and Health Service, Tehran Islamic Republic of Iran. Email: shahrzad.modarres2014@gmail.com , orcid.org/0000-0002-0584-4240.; 4 Department of Biostatics, Shahid Beheshti University of Medical Sciences, Tehran, Iran. Email:alavimajd@gmail.com , orcid.org/0000-0001-7772-2923

**Keywords:** Obstetric, Reliability, Triage, Maternal, Validity

## Abstract

**Introduction:**

The growing demand for high-quality obstetric care and treatment has led to the advent and development of a field known as obstetric triage. The present review study aimed to examine the development of tools and criteria for obstetric triage services.

**Methods:**

In this narrative review, two authors searched for related articles using the keywords of “obstetric triage, gynecology triage, perinatal Triage, maternity triage, midwifery triage” AND “tool, index, scale, questionnaire, system”. With Persian and English language limitation, searches were performed in Scopus, Google Scholar, Scientific Information Database, ProQuest, Medline, Embase and Web of Science databases for articles published from 2000 to 2018.

**Results:**

Out of the 289 articles reviewed in this study, 8 articles met the eligibility criteria. Out of these 8 articles, 6 were dedicated to introducing a tool designed and only 2 introduced an obstetric triage system. The obstetric triage tools and systems covered included Emergency Severity Index (ESI), Obstetric Triage Acuity Scale (OTAS), Birmingham symptom specific obstetric triage system (BSOTS), Maternal Fetal Triage Index (MFTI), Florida Hospital Obstetric Triage Acuity Tool, self-assessment questionnaire for gynecologic emergencies (SAQ-GE) and Perinatal Emergency Team Response Assessment (PETRA). Overall, the validity and reliability of the studied method were investigated and found to be acceptable in only 5 of the reviewed studies.

**Conclusion:**

The review showed the lack of consensus on how to devise a single standardized tool or system for obstetric triage. The comparison of different obstetric triage tools and systems demonstrated the need for a standardized and widely-approved system with high validity and reliability and standard definitions for obstetric triage to determine the right priority and waiting times of obstetric care services.

## Introduction:

Insistent effort to make further improvements in health care systems is a key requirement for achieving the goal of sustainable development in regard to maternal mortality and morbidity (1). The demand for high-quality obstetric care and treatment has led to the advent and development of a field known as obstetric triage (2). Triage is the process of prioritizing patients based on the severity of their condition in order to provide necessary treatments as efficiently as possible in the shortest possible time. Therefore, triage is the basis of care delivery procedure in emergency departments (3, 4). Obstetric triage unit is the place where maternal patients entering the hospital system are initially processed to receive emergency medical and obstetric care (5, 6). Obstetric triage is more specialized than general and trauma triage, as it involves assessing labor condition and fetal well-being and preparing tests and interventions for obstetric problems (7).

The most important issues of obstetric triage are patient dissatisfaction and prolonged waiting times (8-10). A prolonged waiting time means leaving patients without examination, which may result in delayed delivery of necessary care and treatment, patient dissatisfaction, and increased mortality and morbidity (11, 12). Research has shown that reducing the waiting time actually reduces the hospital stay time, lowers the treatment cost, and saves hospital resources (13). Despite these benefits, there is no consensus on the basics of obstetric triage and the rules and criteria that should apply to this procedure (14).

Although the initial assessment of obstetric patients involves typical procedures of checking vital signs, triggers, preventive measures, etc., these procedures are not specifically designed for obstetric triage and emergency conditions (15, 16).

In a study by Angelini et al. (2014), it is stated that there is no standardized and widely-accepted tool for obstetric triage in the United States (5). Hence, there is a need for a collection of credible evidence compiled through systematic examination, combination, and integration of the findings in this field (17). This requires a narrative review of research literature for comprehensive and in-depth examination of the reports regarding the existing obstetric triage tools and systems. Given the importance of the issue and the paucity of such reviews, the present review study was conducted to examine the development of tools and criteria for obstetric triage services.

## Methods:

**Search strategy**

In this narrative review, two authors searched for related articles using the keywords of “obstetric triage, gynecology triage, perinatal triage, maternity triage, midwifery triage” AND “tool, index, Check list, questionnaire, system”. The search strategy was as follows: [Obstetric triage OR Maternity triage OR Gynecology triage OR Perinatal Triage OR Maternity triage AND Tool OR Index OR Check list OR questionnaire OR system]. After completion of the document search, duplicate articles were first removed. Then, the title and abstracts were first assessed by two of the authors and unrelated articles were deleted and then some articles underwent final assessment according to inclusion criteria. 

**Databases accessed**

By setting time and language limitations, a search for Persian and English articles published in the period from 2000 to 2018 was performed in Scopus, Google Scholar, Scientific Information Database, ProQuest, Medline, Embase and Web of Science databases.

**Screening and data extraction**

Documents and books related to scales or systems in obstetric triage were included. Full-text not being accessible, non-relevance to the subject, studies not demonstrating a clear research methodology, conference presentations, case reports, letters to editor, language not being English or Persian, and year of publication of the article outside the considered time interval (2000-2018) were considered as exclusion criteria. 

## Results:

**Figure1:**
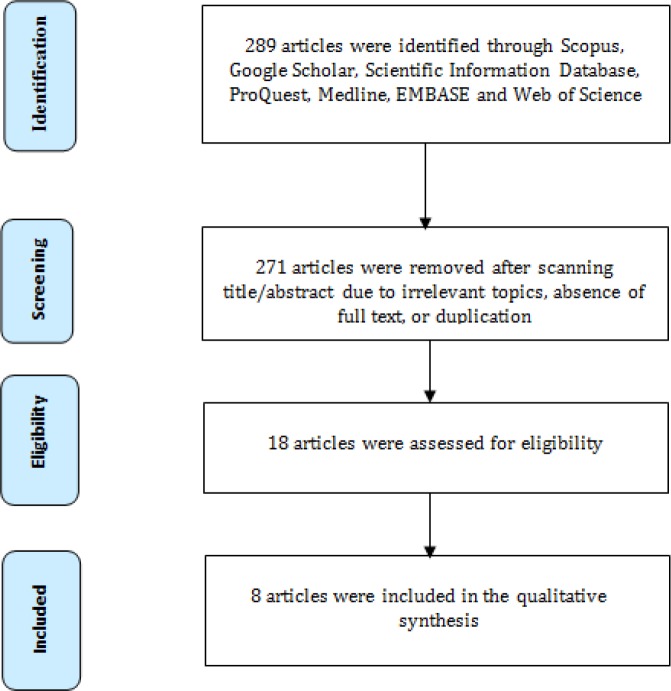
Flowchart of article select

In this study, a total of 289 articles were reviewed. Out of these 289 articles, 18 were found to be eligible for further study. Among them, 8 articles were dedicated to introducing tools and assessing their validity and reliability, 6 introduced a designed tool, and only 2 introduced an obstetric triage system (figure 1).

**Obstetric Triage Acuity Scale (OTAS)**

This scale was originally designed by Smithson et al. (2013) and later expanded by Gratton et al. (2016). OTAS is an obstetric triage scale based on the Canadian Triage Acuity Scale (CTAS), which consists of five levels: critical, emergency, urgent, semi-urgent, and non-urgent (3, 18). The OTAS system also facilitates the assessment of the distribution of acuity and flow and care delivery based on acuity. In this scale, the acuity is color coded. The items considered in this scale include the onset of labor, rupture of fetal membranes, bleeding, hypertension, and fetal assessment.

This tool covers major pain complaints, abdominal trauma, infection symptoms, substance abuse, and psychological problem. In its final form, the tool also covers hemodynamic stability (examination for shock signs, compromise, and abnormal vital signs), respiratory distress, fetal wellbeing, cervical dilation, and vital pregnancy-specific parameters (3, 19, 20). 

In a study by Smithson et al. (2013), they measured the reliability of OTAS and determined the patient flow based on 110 triage charts filled by 8 triage nurses. This study found a kappa value between 0.61 and 0.77 for the first to fourth OTAS levels and a kappa value of 0.87 for the fifth level. They also found that OTAS reduced waiting time (21).

Gratton et al. (2016) also conducted a study on the triage nurses of three hospitals (London Health Sciences Centre, Stratford General Hospital, and Chatham General Hospital) to determine the reliability of OTAS. This study reported that OTAS has significant and comparable inter-rater reliability (IRR) in the studied hospitals and also has significant intra-rater reliability (ITR) (19).

**Swiss Emergency Triage Scale (SETS)**

Designed in 1997 based on the Canadian Acuity and Triage Scale, this tool consists of four levels: immediate-life-threatening, potentially life-threatening, stable situation, and non-urgent situation, which require immediate examination, examination within 20 minutes, examination within 2 hours, and non-urgent examination or referral to clinics, respectively (22).

In a prospective study conducted by Rubin et al. (2017) on 22 midwives and triage nurses in Geneva hospitals, they attempted to determine the psychometric properties of an obstetric triage tool. This study was designed in a two-stage format, which consisted of pre-test and post-test stages with a 6-month interval. The evaluation method involved 30 clinical scenarios designed by a team of experts and a 3D computer simulation. In this study, participants determined the triage based on the Swiss Emergency Triage Scale (SETS). The inter-rater reliability in the first stage (pre-test) was found to be 0.748 (95%CI: 0.653-0.858). In the post-test stage, the inter-rater reliability was calculated to 0.812 (95%CI: 0.726-0.889). Overall, the results showed that SETS has an ICC of 0.7 and can be considered a reliable tool for management of maternal and obstetric emergencies (16).

**Birmingham symptom specific obstetric triage system (BSOTS)**

This system has been designed by a team of researchers and physicians with expertise in obstetrics and gynecology (23). In this system, the clinical indices and related parameters, which have been determined using the Manchester system (24), have been organized in four levels for initial examination and triage. This method of triage involves the assessment of the mother's medical history, vital signs, pains, and fetal heartbeat by a midwife in the presence of a gynecologist. The BSOTS manual for time requirements of care delivery makes use of four colors, red, orange, yellow, and green, which refer to the necessity of providing care immediately, within 15 minutes, within 1 hour, and within 4 hours, respectively. Also, a standardized algorithm has been developed to investigate abdominal pain, gestational bleeding, hypertension, suspected labor, membrane rupture, decreased fetal movement, and postpartum problems.  Kenyon et al. (2017) performed a study to design and implement an obstetric triage system for unwanted pregnancy. In this study, a structured audit was conducted on 994 sets of maternity notes, and reliability evaluation was performed using a scenario-based method. The results showed that the system has excellent reliability in assessing the women’s clinical priority (23).

**Maternal Fetal Triage Index (MFTI)**

Maternal Fetal Triage Index is a clinical tool designed by a team from the Association of Women’s Health, Obstetric and Neonatal Nurses (AWHONN) for standardizing the triage of pregnant women. This tool is an algorithm consisting of five levels: 1-Stat, 2-Urgent, 3-Prompt, 4-No urgent, and 5-Scheduled or Requesting a Service, to which patient will be assigned based on the assessment of their clinical conditions. The Stat level patients require immediate intervention to protect the life of mother or fetus. The Urgent level patients are the people showing clinical conditions at the second level of urgency, such as severe pain without labor and risky clinical conditions, and may require higher levels of care. The Prompt level conditions include the onset of active labor at the gestational age of over 34 weeks or the delay of labor in women undergoing the labor phase. The No-urgent level conditions include the gestational age of 37 weeks, signs of early labor, and common pregnancy complaints. The final level, which is termed Scheduled or Requesting a Service, refers to conditions that can be safely addressed at a later date (25, 26).

In a study by Ruhl et al. (2015), they measured the content validity of MFTI using I-CVI and S-CVI indices. The results of this study showed that MFTI is a reliable tool for triage of pregnant women (26).

Ruhl et al. (2015) also conducted a study on 211 pregnant women to determine the reliability of MFTI. In this study, the minimum reliability of MFTI was measured as 0.60 (27).

**Florida Hospital Obstetric Triage Acuity Tool**

The development of Florida Hospital Obstetric Triage Acuity Tool was first started by Paisley et al. in 2007. This tool is a five-level scale with pregnancy criteria for estimating the examination time requirement based on acuity. The first level (Immediate) covers the conditions that require immediate action such as resuscitation, respiratory distress, chest pain, trauma, bleeding, presenting fetal parts, umbilical cord prolapse, impending delivery, and seizures (28). The second level (Urgent) includes the conditions that should be examined within 15 minutes such as active labor phase, vaginal discharge, preterm labor, spotting, fetal well-being, rupture of fetal membranes, high blood pressure, UTI symptoms, mental disorders, history of epilepsy and diabetes, and pain scores of more than 7. The third level (Semi-Urgent) covers the conditions that must be addressed within 30 minutes, such as R/O labor (irregular uterine contractions at gestational age of more than 37 weeks, mean pain score of 4-6), vaginal discharge, preterm labor at gestational age of more than 37 weeks, fetal well-being, fetal mobility, high blood pressure, mental disorders (with suicide intention or history of suicide attempt), and other factors (repeated C-section, recent trauma due to accident or falling, fever, chills, active nausea, mean pain score of 4-6) (28).

The fourth level (Less-Urgent) includes the conditions that necessitate examination within 60 minutes, such as R/O labor (early labor, mild irregular uterine contractions, back pain at the gestational age of more than 37 weeks, mild pain with mean score of 1-3, vaginal discharge (with blood or mucus, with and without infection), mental disorders (non-OB complaints, insomnia, psychosocial problems, not acting out), and other factors (pain, nausea, gestational vomiting, mild pain with mean score of 1-3).

The fifth level (Procedure/Testing) includes the conditions that necessitate examination within 120 minutes, such as Fetal Well-Being through NST, BPP, ultrasound, other factors (elective C-section, labor induction) and other procedures (incision, breech presentation, betamethasone injection) (28).

In the study of Kathleen et al. (2011), where they created an obstetric triage tool, the results showed that patients were examined within the specified time based on the acuity of their condition (28).

**Self-assessment questionnaire for gynecologic emergencies (SAQ-GE)**

SAQ-GE has been developed using qualitative methods through consultation with a team of French experts. This tool consists of 89 items in six categories: qualitative description of pain, intensity of pain, location of pain, time-course of pain, vaginal bleeding, and other signs (29). 

Huchon et al. (2014) conducted a cohort study to evaluate the performance of SAQ-GE for the triage of potentially life threatening emergencies (PLTE) among women. Out of the 574 eligible patients who completed the SAQ-GE form, 516 entered the study. The results of this study showed that the triage based on a standardized questionnaire facilitates the early diagnosis of patients with PLTEs (29).

**Perinatal Emergency Team Response Assessment (PETRA)**

PETRA is a non-technical skill group assessment tool consisting of seven main categories, namely mental model, communication, situational awareness, leadership, followership, workload management, and positive/effective behaviors and attitudes, which are scored based on 5-point Likert scale.

To assess the validity of PETRA, Balki et al. (2017) conducted an observational cohort study on 119 people in Toronto. The results showed that PETRA is easy to understand (80% agreed, 20% somewhat agreed) and easy to use (60% agreed, 40% somewhat agreed) (30). 

## Discussion:

The present study was a narrative review of obstetric triage systems and tools. Overall, this review showed that the reliability values reported in the five studies on this subject are acceptable.

While the structure of OTAS and MTFI both consists of five levels, BSOTS and SETS have a four-level structure. In the five-level MFTI and Florida, the recommended examination times are 0 minutes (immediate), 10 minutes, 30 minutes, 60 minutes and 120 minutes. But the examination times in the five-level Florida tool are 0 minutes (immediate), 5-15 minutes, 15-45 minutes, 1-2 hours, and 4 hours, and in BSOTS these times are 0 minutes (immediate), 15 minutes, 60 minutes, and 240 minutes.

In the systematic review by Angelini et al. (2014) with the aim of assessing obstetric triage in the past fifteen years, the results showed that the best model in obstetric triage is a model with use of a tool specific for obstetric triage, standardization of assessments, identification of challenges, team training, quality improvement, competent staff, assessment of patient flow with acuity distribution, create a fast track unit, development of clinical protocols in accordance with the rules and regulations (5).

A standardized obstetric triage tool may provide the means for better examination of the care quality and the triage of pregnant mothers and their fetus (31).

Any comparison of different obstetric triage tools should take into account the fact that underlying differences may affect the results of triage (32). The basic prerequisites for determination of care delivery priority and waiting time are access to well-outlined and standardized definitions for obstetric triage and the validity and reliability of the tool used for this purpose (26).

According to Angelini et al. (2014), there is no standardized and widely-approved tool for obstetric triage in the United States (5). Therefore, the validity and reliability of determinants of patient priority in maternal and fetal care cases should be further investigated (5).

Paisley et al. (2011) have shown that in the absence of a well-defined triage system, patients who fall in the second and third levels of obstetric triage will not be examined in due time, and instead, patients in the fourth and fifth levels will receive care earlier. Hence, proper implementation of an obstetric triage tool is of immense clinical importance (28).

***Limitations***

The limitations of this study included the heterogeneity of the methods adopted in the reviewed studies for reporting the variables of interest and the lack of access to all of the related documents published worldwide as well as gray literature. Further research on the existing general triages with potential application in obstetrics is recommended.

## Conclusion:

The review showed the lack of consensus on how to devise a single standardized tool or system for obstetric triage. The comparison of different obstetric triage tools and systems demonstrated the need for a standardized and widely-approved system for determining the proper priority and waiting times of obstetric care services, with high validity and reliability and standard definitions for obstetric triage.
